# Ensheathing cells utilize dynamic tiling of neuronal somas in development and injury as early as neuronal differentiation

**DOI:** 10.1186/s13064-018-0115-8

**Published:** 2018-08-18

**Authors:** Ev L. Nichols, Lauren A. Green, Cody J. Smith

**Affiliations:** 1 Department of Biological Sciences, University of Notre Dame, 015 Galvin Life Sciences Building, Notre Dame, IN 46556 USA; 2 Center for Stem Cells and Regenerative Medicine, University of Notre Dame, Notre Dame, IN USA

**Keywords:** Development, Ensheathment, Neuronal soma, Tiling, Neural niche

## Abstract

**Background:**

Glial cell ensheathment of specific components of neuronal circuits is essential for nervous system function. Although ensheathment of axonal segments of differentiated neurons has been investigated, ensheathment of neuronal cell somas, especially during early development when neurons are extending processes and progenitor populations are expanding, is still largely unknown.

**Methods:**

To address this, we used time-lapse imaging in zebrafish during the initial formation of the dorsal root ganglia (DRG).

**Results:**

Our results show that DRG neurons are ensheathed throughout their entire lifespan by a progenitor population. These ensheathing cells dynamically remodel during development to ensure axons can extend away from the neuronal cell soma into the CNS and out to the skin. As a population, ensheathing cells tile each DRG neuron to ensure neurons are tightly encased. In development and in experimental cell ablation paradigms, the oval shape of DRG neurons dynamically changes during partial unensheathment. During longer extended unensheathment neuronal soma shifting is observed. We further show the intimate relationship of these ensheathing cells with the neurons leads to immediate and choreographed responses to distal axonal damage to the neuron.

**Conclusion:**

We propose that the ensheathing cells dynamically contribute to the shape and position of neurons in the DRG by their remodeling activity during development and are primed to dynamically respond to injury of the neuron.

**Electronic supplementary material:**

The online version of this article (10.1186/s13064-018-0115-8) contains supplementary material, which is available to authorized users.

## Background

Ensheathment of neuronal cells is essential for proper functioning of the nervous system. The role of ensheathment is diverse and ranges from functions such as myelination to aid neural transmission [1–3], to metabolic homeostasis and trophic support to maintain neuronal health [4, 5]. This ensheathment typically occurs at an early, yet largely undefined, time in the development of functional neural circuits and is consistently maintained following the initial ensheathment [3, 6, 7]. Disruption of ensheathment at any point can result in various pathologies depending on the cell types affected [5, 8]. Axonal ensheathment and myelination have been extensively studied, but an understanding of neuronal soma ensheathment in vertebrates remains elusive [1, 9, 10].

Studies in *C. elegans* and *Drosophila* have observed that glial subpopulations extend processes around the soma to completely ensheath the neuron [11, 12]. These soma-ensheathing glia play an important role in development. Studies in *C. elegans* demonstrate that glial association with neurons guides neuronal maturation and axonal outgrowth [13–15]. These ensheathing glia also are closely associated with the neurons and respond to changes within the cell [11]. For example, after neuronal injury these glia have been shown to respond and phagocytize debris [16]. Previous work also demonstrates that disruption of cortex glia, the cells ensheathing neuronal cell somas in *Drosophila*, leads to neuronal death, suggesting an important role of ensheathing glia in neuronal growth and survival [17]. Disruption to cortex glia also leads to the disorganization of neuronal and glial cell populations in the *Drosophila* CNS [17, 18]. In vertebrates, molecular communication between the neuronal soma and oligodendrocytes ensures the proper ensheathment of specific neuronal compartments, like axons [19]. Collectively, these studies point to tightly controlled and conserved interactions between ensheathing glia and sub-compartments of neurons on the macroscopic and microscopic level.

One organizational principle of glia that has emerged is cellular tiling. Tiling ensures comprehensive yet non-redundant coverage of a target area [11, 20, 21]. This principle appears to be largely universal across cell-types and phylogeny as retinal neurons [22], sensory neurons [20, 21, 23–25], oligodendrocytes [26, 27] and other ensheathing glia [17, 28] have all demonstrated tiling in numerous model systems. In glial cells, this tiling behavior has been shown to subdivide neural domains by creating a comprehensive sheathe around the target area without redundant commitment of cellular resources [29]. Disruption of glial tiling has been shown to disrupt the overall organization of neural compartments [17, 18, 30]. A hallmark of tiling is a space filling response from neighboring cells following perturbation [7, 27, 31]. For example, oligodendrocyte progenitor cells have been reported to exhibit tiling behavior following injury to mature oligodendrocytes [27]. During development, oligodendrocytes also exhibit tiling to space themselves in the spinal cord [26, 27]. Oligodendrocytes also tile with other cell types in the nervous system [28]. This tiling phenomenon with glia is likely more extensive given that both Drosophila astrocytes in the brain and cortex glia around cell somas in the CNS also exhibit tiling [18, 29]. However, the role of tiling in other glia, like those that ensheath neuronal cell somas has yet to be described in vertebrates.

During early development, neuronal populations are often associated with glial progenitors that tightly ensheath the differentiating neuron and its progenitors [32]. This ensheathment of progenitor populations has been shown to aid in strict compartmentalization of the nervous system which helps give rise to stereotyped neuronal topographies such as *Drosophila* brain lobes [11, 17, 30, 32]. The dorsal root ganglia of vertebrates (DRG) also exhibits a stereotyped sequestration of neuronal subtypes in a specific spatiotemporal manner. Mechanoreceptive and proprioceptive neurons develop first in the ventrolateral region of the DRG followed by nociceptive neurons in the dorsomedial region of the ganglion [33]. The DRG is also home to a subset of glial cells that ensheathe DRG neuronal somas: satellite glia [9, 10]. Throughout life, these cells serve as important precursors for a diverse set of cell types such as terminal glia and melanocytes that locate to the periphery [34]. During differentiation of these cells, resident precursors in the DRG must migrate through the packed ganglia, exit and then travel along nerves to the periphery. This process of differentiation continues through adulthood.

It is currently unclear if and how satellite glia actively and continually contribute to the stereotyped topography of the DRG. However, genetic ablation of glial precursors or disruption of precursor migration to the nascent DRG has been shown to disturb proliferation and cell fate decisions, leading to an disorganized or absent DRG [23, 35, 36]. DRG neurons can also be ectopically located outside of their normal ganglia location when non-neuronal ganglia cells are disrupted [23]. These studies highlight the possibility that cellular mechanisms are constantly and dynamically employed within the ganglia to ensure neurons are stereotypically located while non-neuronal populations retain the capacity to translocate when needed.

Our understanding of these topics is limited because techniques to dynamically image and label glial progenitors distinctly from the neuron that they ensheath has been lacking. Moreover, the majority of studies that investigate ensheathment of neuronal cell somas has typically investigated the ensheathment of fully differentiated neurons. To address this, we used single cell photoconversion of glial progenitors within the nascent DRG in zebrafish. Using this approach, we visualized cells extending pericellular processes around neuronal progenitors. These sheaths were present throughout the life of the DRG, including during neurite initiation where they remodel to allow for neurite extension. We demonstrate these ensheathing cells exhibit cellular behaviors consistent with dynamic tiling in response to disruption of ensheathment of the neuronal soma in both normal development and disease states. Such pathological disruptions to the neuronal soma ensheathment also led to immediate changes in neuron size and shape, suggesting they could play an active role in restricting neuronal positioning in the ganglia. These ensheathing cells also exhibited a consistent and choreographed response to peripheral axonal injury. These data provide an important step-wise visualization of the neuronal soma ensheathment early in development and provide insight into how cell soma ensheathing glia dynamically ensure DRG neurons retain their stereotypical ganglia location.

## Methods

### Fish husbandry

All animal experiments were approved by the University of Notre Dame Institutional Animal Care and Use Committee. The zebrafish transgenic lines used in this study were *Tg(sox10:eos)* [37]*, Tg(ngn1:gfp)* [38]*,* and *Gt(foxd3:gfp)* [39]*.* All embryos were produced by pairwise mating and raised in egg water at 28 °C. Embryos of both sexes were used for all experiments. Stable, germline, transgenic lines were used for all experiments.

### In vivo imaging

All embryos were dechorionated at 48 hpf and anesthetized with 3-amino-benzoic acid ester. Anesthetized embryos were mounted in 0.8% low-melting point agarose and mounted on their right side in 35 mm Petri dishes with glass bottoms. For imaging, a spinning disk confocal microscope from 3i technology© was used. It is equipped with a Zeiss Axio Observer Z1 Advanced Mariana Microscope with X-cite 120LED White Light LED System and filter cubes for GFP and mRFP, a motorized X,Y stage, piezo Z stage, 20X Air (0.50 NA) objective with 2 mm working distance, 63X (1.15NA) water objective with 0.66 mm working distance, 40X (1.1NA) water objective with 0.62 mm working distance, CSU-W1 T2 Spinning Disk Confocal Head (50 uM) with 1X camera adapter, andor iXon3 1Kx1K EMCCD camera, dichroic mirrors for 446, 515, 561, 405, 488, 561, 640 excitation, laser stack containing 405 nm, 445 nm, 488 nm, 561 nm and 637 nm with laserstack FiberSwitcher that has 250 uS switch time, photomanipulation with vector© high speed point scanner ablations at diffraction limited capacity, Ablate!TM© Photoablation System (532 nm pulsed laser, pulse energy 60 J @ 200 HZ). Time lapse images were taken every 5 min over 24 h. Adobe Illustrator and ImageJ were used to process the images and enhance the brightness and contrast.

### Eos photoconversion

To label individual progenitor cells, we used *Tg(sox10:eos)* fish to express Eos, a photoconvertible protein in DRG cells. Upon exposure to ultraviolet light, Eos transitions from green to red emission. Using the laserstack system described above, single cells were exposed to a 405 nm laser through the z-stack [40]. This laser exposure resulted in the photoconversion of the targeted cell from green to red. Pre and post images were acquired to ensure photoconversion of single cells within the DRG was accomplished.

### Immunohistochemistry

Zebrafish were fixed and stained as previously reported [28]. Primary antibody Sox10 (rabbit, 1:5000) [28] and secondary antibody Alexa anti-mouse conjugated to 561 (Invitrogen, 1:600) was used. DAPI was also used (ThermoFisher, 1:1000).

### Focal lesioning

To create lesions of individual cells and axons, the Ablate!TM© Photoablation System described above was utilized. Adjacent, unablated DRG or axons were used as controls. One lesion at a DRG was performed in each animal. Following the lesion, time lapse images were taken as described above. Animals were placed 0.02% 3-aminobenzoic acid ester (Tricaine) in egg water with anesthetic and mounted with 0.8% low-melting point agarose on a 10 mm glass-coverslip bottom petri dish. Confocal z-stack images of *Tg(sox10:eos)* at the appropriate age were taken pre-injury. A DRG cell or axon was chosen and brought into a focused ablation window. To ablate we utilized a double-clicked feature which creates an 8 um cursor tool to fire the ablate laser. All laser parameters used are specific to our confocal microscope. Specific parameters include: Laser Power (2), Raster Block Size (4), Double-Click Rectangle Size (8), Double-Click Repetitions (4).

### SU6656 treatment

A stock solution of SU6656 (Santa Cruz Biotechnology) was stored at − 20 °C at a concentration of 375 μM in DMSO. Treated embryos were manually dechorionated at 36 hpf and incubated at 28 °C in 3 μM of SU6656 in egg water until imaging as previously described [41]. Control fish were incubated in 1.25% DMSO in egg water.

### Shape descriptors

The shape descriptors (circularity, aspect ratio, roundness, and solidity) were measured using ImageJ. They were calculated using the following formulas:

$$ Circularity=\frac{4\pi \times area}{(perimeter)^2} $$

$$ Aspect\ Ratio=\frac{major\ axis}{minor\ axis} $$

$$ Roundness=\frac{4\times area}{\pi \times {\left( major\ axis\right)}^2} $$

$$ Solidity=\frac{area}{convex\ area} $$

### Quantification of approaching ensheathing processes

**Fig. 1:**
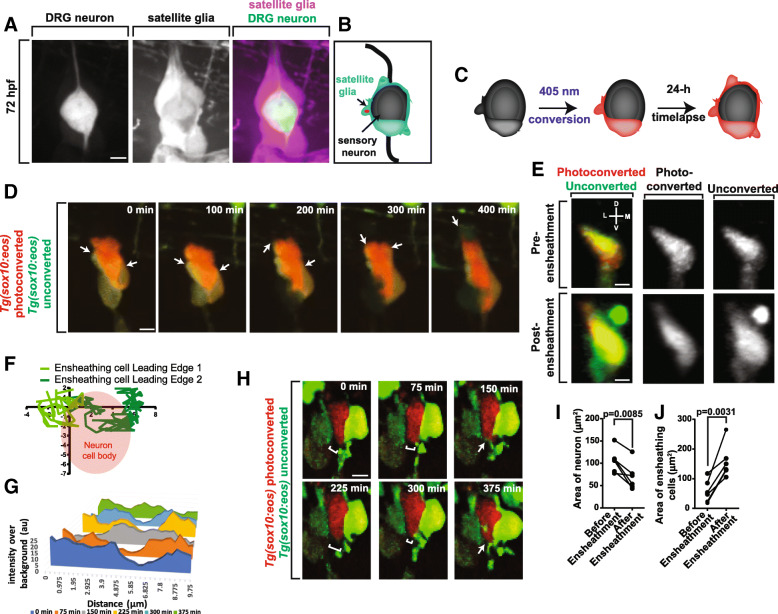
Sensory neurons become ensheathed shortly after neuronal differentiation. (**a**). Confocal z-projection frame of a *Tg(sox10:mcherry); Tg(ngn1:gfp)* zebrafish DRG at 72 hpf showing complete ensheathment. (**b**). Diagram of sensory neuron cell soma ensheathment by satellite glia. (**c**). Diagram of Eos photoconversion paradigm before neuronal differentiation. (**d**). Confocal z-projection images from a 24-h timelapse starting at 48 hpf of a *Tg(sox10:eos)* zebrafish with a photoconverted DRG neuron showing ensheathment of neuronal cell soma. White arrows denote dynamic projections of the ensheathing cell circumnavigating the neuron soma. (**e**). Confocal three-dimensional images of a nascent *Tg(sox10:eos)* DRG with a photoconverted neuron before and after ensheathment. D denotes dorsal, M denotes medial, V denotes ventral, and L denotes lateral. (**f**). Plot of two ensheathing processes converging on the same area of the of the neuron. (0,0) represents the site of the convergence of ensheathing processes. Red circle denotes the location of the neuron cell soma. (**g**). Intensity profiles transecting two approaching ensheathing processes every 75 min. (**h**). Deconvolved confocal z-projection images of two approaching ensheathing projections represented in (**g**). White brackets denote the gap between the two ensheathing processes. White arrows denote the emergence of the cellular processes. (**i**, **j**). Graphs of the areas of neurons (**i**) and ensheathing cells (**j**) before and after neuronal soma ensheathment (*n* = 5 DRG). (**i**, **j**) use a paired Student’s *t-*test. Scale bar is 10 μm (**a**, **d**, **e**, **h**). All intensity measurements were taken from sum z-projections

To create the intensity profiles found in Figs. 1g and 4b, sum z-projection frames from a 24-h timelapse were deconvolved using Autoquant Blind deconvolution in Slidebook software. Intensity profiles were created along a line that transected two approaching processes. These profiles were taken at 75 min intervals as the cellular processes approached to make a coherent sheathe around the neuron soma. All intensity values were normalized to the background intensity of the image. These graphs quantify the ensheathment of a single neuron.

### Quantification and statistical analysis

Slidebook software was used to create maximum and sum z-projections for all images used for analysis. Individual z-projections were sequentially reviewed to confirm accuracy. All data presented in graphs represent the mean of data. All error bars represent standard error of the mean. Cell tracking was performed using MTrackJ, a plugin for ImageJ. All intensity measurements were taken from sum Z-projections and were normalized to background intensity. GraphPad Prism and Microsoft Excel were used to create all graphs and perform statistical analyses. Unpaired Student’s *t-*tests, paired Student’s *t-*test, and unpaired one-way ANOVAs with multiple comparisons (Tukey’s Honest Significant Difference test) were used to calculate statistical significance as noted in the figure legends.

## Results

Differentiated dorsal root ganglia neurons (DRG) are encased by a perineuronal glial population known as satellite glia [9, 10]. We visualized this ensheathment in zebrafish using transgenic animals that express a fluorescent protein in glial populations using *sox10* regulatory sequences and expression of a different reporter in neuronal cells using *neurogenin1*, *ngn1*, regulatory sequences (Fig. 1a,b). We sought to identify how the progenitor populations envelop the neurons during the initial development of the neuron when it is morphologically plastic.

### Neurons are ensheathed during early neuronal differentiation

To dissect how these ensheathing cells develop in relation to the developing neuron, we devised an experimental paradigm that allowed us to mark individual cells of the nascent DRG before they expressed their differentiation markers. To do this we utilized *Tg(sox10:eos)* animals which express the photoconvertible protein, Eos, in DRG precursors under the regulatory sequence of *sox10* [37]. To label individual DRG cells we photoconverted single cells within the nascent DRG by directing UV laser light with our confocal microscope system to an 8 μm region in each DRG [40]. Since a typical DRG cell at this age in development is approximately 10 μm in diameter we could reliable photoconvert single cells within the DRG (Fig. 1c). Using this setup, we then could collect confocal z-stacks spanning the DRG every 5 min for 24 h and produce of movie of early DRG development. In zebrafish at 48 h post fertilization (hpf), the DRG is comprised of 2–4 cells.

We first hypothesized that the ensheathing progenitor would extend processes around the cell membrane of the developing neuron, like reported in axonal ensheathment by Schwann cells [42]. We further hypothesized that cell soma ensheathment likely occurs after axonal extensions are completed. To test these hypotheses we imaged *Tg(sox10:eos)* animals every 5 min for 24 h from 48 to 72 hpf. At the start of these movies, we could reliably visualize two round cells within the ganglia (Fig. 1d, Additional file 1: Movie S1). Using cell tracking software, we determined that after 74.6 ± 21.4 min, one cell remains round (neuronal progenitor) and the other cell within the DRG extends cellular processes that guide along the edge of the other DRG cell (*n* = 12 ensheathing processes). These processes move at an average speed of 0.029 ± 0.0083 μm/min (n = 12 processes). The cellular extensions eventually encircle the entire round DRG cell completing the initial ensheathment of that cell. After ensheathment, the round DRG cell that is ensheathed adopts a more amoeboid-like morphology but continues to be enveloped by the other DRG cell even at these early stages (Fig. 1d,e). Within 24 h, the ensheathed cell produces a dorsally and ventrally projecting axon indicating its neuronal identity. We hypothesized that this ensheathment was occurring around the entire neuronal progenitor rather than just encircling it in a single dimension. To dissect this possibility, we rotated the images of the nascent DRG 90° before and after ensheathment so that the x-axis of the image would represent the lateromedial axis of the animal (Fig. 1e). These images show that the ensheathing progenitor completely surrounds the neuronal soma in all three dimensions. Taken together, these results are consistent with hypothesis that the neuron is ensheathed even before neuronal projections are produced, indicating that they are ensheathed throughout the neuron’s lifespan.

We next sought to gain a step-wise understanding of neuronal soma ensheathment. We hypothesized that this process is similar to axonal ensheathment where membranous sheets extend around the axon [19]. Alternatively, thin, finger-like projections could be initiated by the ensheathing progenitors, more similar to astrocyte processes [43]. To test this hypothesis, we used cell tracking software to determine the dynamic arrangement of ensheathing cells around the soma. In this analysis, we identified that early ensheathment occurs as two pericellular extensions independently migrate around the neuronal precursor until they converge and the entire soma is covered by a membrane. Figure 1f represents the ensheathment dynamics of a representative DRG by two distinct pericellular extensions. The ensheathing extensions travel variable speeds and total distances, as much as 55.6 μm or as little as 3.1 μm (*n* = 6 DRG, 12 ensheathing processes, mean ± SEM: 25.9 ± 6.3 μm). Ensheathing projections that traveled shorter distances still ensured that the entire round neuronal cell is covered by cell membrane. We did not observe the finger-like projections in this analysis but rather the extension of a migrating leading edge around the soma. These results are consistent with the model that neuronal ensheathment occurs by ensheathing cells initiating projections that wrap around the entirety of the neuronal soma.

Given that multiple projections lead to the ensheathment of the neuronal soma, we also sought to determine how a consistent ensheathment around the neuron is achieved. We hypothesized that the pericellular processes could: 1. halt their navigation immediately upon contact with a neighboring ensheathing cell, 2. overlap to form layers of ensheathing cells in the ganglia, or 3. stabilize with a gap between them. To distinguish these possibilities, we measured intensity profiles at 75 min intervals that transect the two ensheathing projections as they approach each other (Fig. 1g) as has previously been completed for oligodendrocyte sheathe formation [3]. If cellular processes overlapped, then we would expect a sharp increase (~ 2×) in the fluorescent intensity at the point of intersection. If there was a gap between cellular processes, then decreased intensity would be detected at the center point of the transection. Our data reveal that after two projections approach the same area, the level of fluorescence was measured as a single uniform platform that was not significantly larger than the individual extending peaks, consistent with the hypothesis that two projections could contact each other without measurable overlap. Additional time points showed a decrease in florescent intensity into two distinct platforms, consistent with a slight retraction of the processes of approximately 2.28 μm. Eventually, in all 5 DRG that were analyzed, the profiles return to a single platform (Fig. 1g,h). Unfortunately, because of the spatial resolution limits of light microscopy, homotypic contact cannot be definitively concluded. However, together these data are consistent with the hypothesis that ensheathing cells likely utilize a self-correction mechanism to completely, but non-redundantly cover their target – the neuronal cell soma.

To investigate if, in addition to changes in the ensheathment event, the neuronal soma also changed shape during ensheathment, we measured the area of the glia and neuron both before and after the ensheathment of the neuron. These measurements revealed that the glia greatly expand in size during ensheathment consistent with their organization to completely envelop the neuron (Fig. 1j, *p* = 0.0031, *n* = 6, mean difference ± SEM: 100.6 ± 18.91 μm^2^). Conversely, the neuron decreases in area during ensheathment (Fig. 1i, *p* = 0.0085, n = 6, − 36.07 ± 8.6 μm^2^). These data raise the possibility that early ensheathment event of the DRG neuron may be important for restricting the size, shape, and/or location of the developing neurons in the ganglia.

### Ensheathing progenitors remodel to cover neurons during neurite extension

**Fig. 2:**
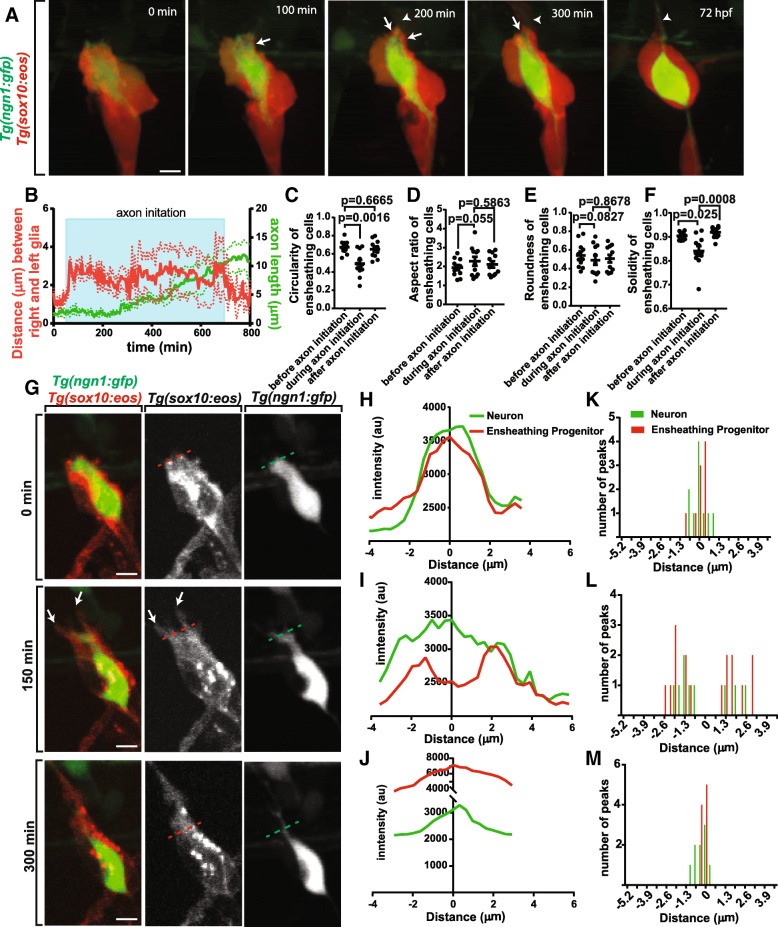
Ensheathing cells remodel during neurite outgrowth. (**a**). Confocal z-projection images from a 24-h timelapse starting at 48 hpf of *Tg(ngn1:gfp), Tg(sox10:eos)* zebrafish with photoconverted ensheathing cells. White arrowheads denote the extending axon. White arrows denote glial horns. (**b**). Graph of the distance between the two dorsally located ensheathing projections (red) and the length of the extending axon (green). Shaded blue box denotes the period of axon initiation (*n* = 12 DRG). (**c**-**f**). Graphs of the circularity (**c**), aspect ratio (**d**), roundness (**e**), and solidity (**f**) of the ensheathing glia before, during, and after axon initiation (n = 12 DRG). (**g**). Confocal images of a *Tg(ngn1:gfp), Tg(sox10:eos)* DRG before, during, and after axonal initiation showing glial horn formation. Dashed red and green lines denote the transecting lines used for the intensity profiles in (**h**-**j**). (**h**-**j**). Intensity profiles of *Tg(ngn1:gfp)* expression (green) and photoconverted *Tg(sox10:eos)* expression (red) through the dorsal apex of the DRG neuron before (**h**), during (**i**), and after (**j**) axon initiation. The line x = 0 represents the center of the neuron. (**k**-**m**). Histogram of the location of the *Tg(ngn1:gfp)* (green) and photoconverted *Tg(sox10:eos)* (red) intensity peaks before (**k**), during (**l**), and after (**m**) axon initiation (n = 12 DRG). X = 0 represents the center of the neuron. (**c**-**f**) use a Tukey’s honest significant difference (HSD) test. Scale bar is 10 μm (**a**, **g**)

Given that the DRG neuron must extend processes to the periphery and spinal cord, the complete ensheathment of the soma needs to change during the extension of these projections [44]. We reasoned that the ensheathing cells would rearrange to allow for this process to occur. To test this hypothesis we visualized DRG neuronal differentiation using *Tg(ngn1:gfp)* [38] in combination with *Tg(sox10:eos)*. In these animals, GFP is expressed in newly differentiated neurons while Sox10 is expressed in DRG progenitor cells that ensheath the neurons. To distinguish the two transgenes we photoconverted Eos during each 5 min interval. We first hypothesized that the glia could undergo cell death to allow for process extension to occur. However, in our movies we did not visualize cell death during axonal extension. We therefore hypothesized that ensheathing cells dynamically remodel during axonal initiation. To test this we scored the rearrangement of ensheathing cells during axonal extension. In these movies, we visualized that these early neurons extend into an oval-like morphology before initiating an axon that extends dorsally toward the dorsal root entry zone (DREZ). As this extension is initiated, the ensheathed cell/s retracts two projections at the dorsal apex of the neuron (Fig. 2a). To measure this, we used cell tracking software to measure the location of these two projections at 5 min intervals. This allowed us to determine the distance between them throughout initiation of the axonal projection. These calculations revealed an increase in the distance between the two ensheathing projections (~ 3 μm) during the period of axonal initiation (Fig. 2b, *n* = 12). We were also able to measure the length of the extending axon at each time point. These tracings reveal that length of the axon remains constant at 1.5 ± 0.68 μm for the initial period that the ensheathing cell rearranges and quickly increases after approximately 250 ± 90.8 min of ensheathing cell separation (Fig. 2b).

To examine the observed changes in the orientation of the DRG cells during the extension of the axon, we used four measures of shape: circularity, aspect ratio, roundness, and solidity. These measurements were taken of the shape of the ensheathing glia before, during, and after neurite extension. Circularity reflects how closely the shape reflects that of a perfect circle, so a mature, round neuronal soma would have a high circularity value. The circularity of the glia decreases during the process of neurite initiation (Fig. 2c, *p* = 0.0016, n = 12, before: 0.671 ± 0.0214, during: 0.492 ± 0.0425) and then increases to the original level after the extension of the projection is completed (*p* = 0.6665, n = 12, after: 0.652 ± 0.0289). This decrease in glial circularity during axonal extension reflects an irregularly shaped ensheathing glia to allow for the physical extension of neuronal processes. Aspect ratio and roundness are measures of the elongation of a shape. Both the aspect ratio (Fig. 2d, *p* = 0.055, n = 12, before: 1.946 ± 0.139, during: 2.278 ± 0.240, after: 2.113 ± 0.173) and roundness of the ensheathing cells (Fig. 2e, *p* = 0.0827, n = 12, before: 0.542 ± 0.0406, during: 0.490 ± 0.0501, after: 0.507 ± 0.0410) do not change during neurite extension. Last, solidity measures how symmetric and regular a shape is. During rearrangement of ensheathing cells during neurite extension, the solidity of the ensheathing cells decreases (Fig. 2f, *p* = 0.025, n = 12, before: 0.904 ± 0.00486, during: 0.841 ± 0.0203) and returns to a more solid shape following axon initiation (*p* = 0.0008, n = 12, after: 0.920 ± 0.00704). Taken together these data support the hypothesis that the ensheathing cells remodel during neurite extension which has lasting changes on the shape of the neuronal/ensheathing glia unit. These data demonstrate that ensheathment remains plastic as differentiation continues to ensure axonal projections can extend to their appropriate targets.

Previous studies have shown that pioneer axons are associated with Sox10^+^ cells throughout their navigation [45]. To dissect the mechanistic relationship between ensheathment of the neuronal cell soma and association with growing axons we tracked the spatial relationship of the ensheathing cell with the extending nascent axon. We hypothesized that the soma ensheathing cells could rearrange their processes to extend with nascent axons. Conversely, cell divisions could produce a new ensheathing cell/s that associate with the axon. When distinguishing between these possibilities, we often visualized a rearrangement of the soma ensheathing processes where cells parted for neurites to extend resulting in two glial protrusions on each side of the extending axon (Fig. 2g). These protrusions are reminiscent “glial horns.” Given this consistent morphology, we hypothesized that the extending axon may interact with one or both of these glial horns. To explore the potential relationship between the axon and the glial horns, we scored the interaction of the extending neurite with the glial horns by measuring intensity profiles for *Tg(ngn1:gfp)* and converted *Tg(sox10:eos)* that transected the two glial projections before, during, and after axon initiation. These profiles revealed that before and after axon initiation, the *Tg(ngn1:gfp)* and converted *Tg(sox10:eos)* intensities were represented with a single peak, suggesting that the glia are ensheathing the neuronal soma before and after neurite initiation (Fig. 2h,j). During the initiation of the axon, there are two peaks of converted *Tg(sox10:eos)* intensity, consistent with the observation that the ensheathing cells retract and extend two horns during axon initiation (Fig. 2i). In these profiles, the *Tg(ngn1:gfp)* intensity formed a single peak that was always associated with one of the converted *Tg(sox10:eos)* peaks. To ask if there was a preferential association with one of the glial horns we scored the axons that associate with either horn (Fig. 2k-m). However, these nascent axons did not show a preference for either the anterior or posterior glial horn. Interestingly, nascent axons initially extended and retracted along both horns before ultimately selecting a horn to extend along. The other, adjacent horn without an axon, then returned to the neuronal cell body. Taken together, these data are consistent with the hypothesis that the retraction of the ensheathing glial projections not only physically allows for axon initiation but may also provide a substrate for the developing neurite to stabilize on as it grows.

### Ensheathment of the cell body is independent of axonal ensheathment

With our data that the ensheathing cell of neuronal soma projects dorsally during axonal initiation, we sought to test whether inhibiting the ensheathment of the axon would affect the ensheathment of the cell soma. To do this, we utilized a drug, Src-family kinase inhibitor SU6656, that inhibits the ensheathment of the axonal projection (Nichols and Smith, unpublished data).

**Fig. 3:**
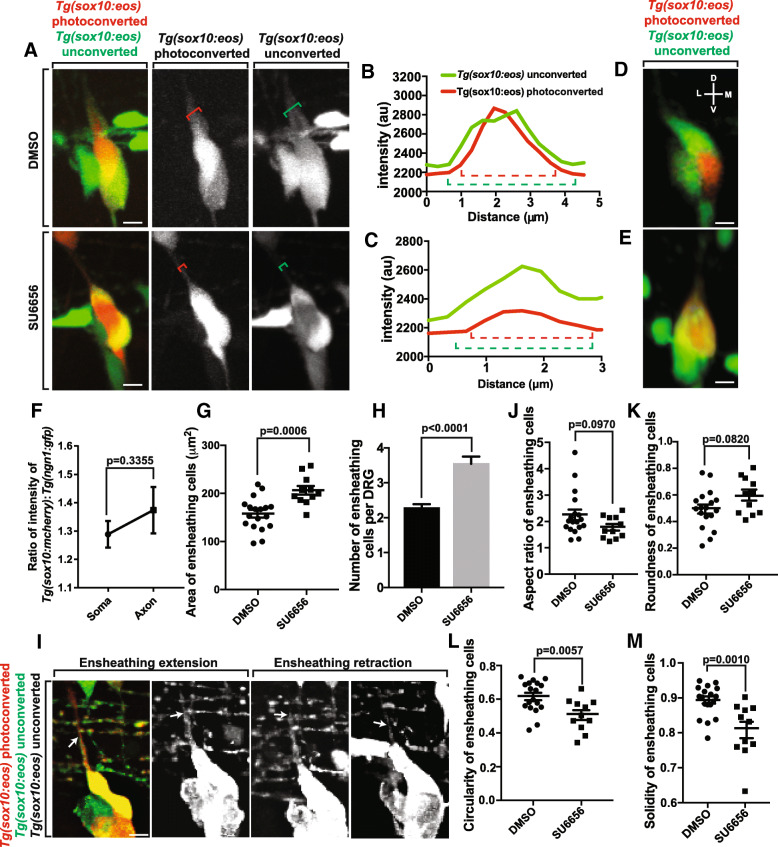
Neuronal cell soma ensheathment is distinct from axonal ensheathment. (**a**). Confocal z-projection images of a *Tg(sox10:eos)* zebrafish with a photoconverted neuron at 72 hpf. The top images were taken of an animal treated with DMSO. The bottom images were taken from an animal treated with SU6656. Red bracket denotes the width of the axon. Green bracket denotes the width of the ensheathing cells. (**b**, **c**). Intensity profiles of *Tg(sox10:eos)* unconverted (green) and *Tg(sox10:eos)* converted (red) transecting the axon from animals treated with DMSO (**b**) and SU6656 (**c**). Dashed red bracket denotes the width of the axon. Dashed green bracket denotes the width of the ensheathing cell. (**d**, **e**). Confocal three-dimensional images of a *Tg(sox10:eos)* DRG with a photoconverted neuron in DMSO and SU6656 treated animals. D denotes dorsal, M denotes medial, V denotes ventral, and L denotes lateral. (**f**). Ratio of the width of *Tg(sox10:mcherry); Tg(ngn1:gfp)* peaks taken from intensity profiles transecting the neuronal soma and axon (*n* = 10 DRG). (**g**) Area of the ensheathing cells in DMSO and SU6656 treated animals (*n* = 18 DMSO DRG, *n* = 11 SU6656 DRG). (**h**). Number of ensheathing cells per DRG at 72 hpf (n = 18 DMSO DRG, n = 11 SU6656 DRG). (**i**). Deconvolved confocal z-projections of a *Tg(sox10:eos)* DRG with a photoconverted neuron in a SU6656-treated animal. White arrow denotes the dorsal tip of an ensheathing cell on the DRG axon. (**j**-**m**). Graphs of the aspect ratio (**j**), roundness (**k**), circularity (**l**) and solidity (**m**) of the ensheathing cells before, during, and after axon initiation (n = 18 DMSO DRG, n = 11 SU6656 DRG). (**g**, **h**, **j**-**m**) use an unpaired Student’s *t*-test. (**f**) uses a paired Student’s *t-*test. Scale bar is 10 μm (**a**, **d**, **e**, **i**). All intensity measurements were taken from sum z-projections

We hypothesized that axonal and neuronal ensheathment were two, distinct processes. However, if the cells that ensheath the axon originate as an ensheathing cell of the neuronal soma, disruption of axonal ensheathment could affect neuronal cell soma ensheathment. To test this, we molecularly manipulated axonal ensheathment and measured neuronal soma ensheathment. We treated *Tg(sox10:eos)* larvae with SU6656 or DMSO at 36 hpf and imaged them from 48 to 72 hpf, the period when axons navigate dorsally and become ensheathed (Fig. 3a). To distinguish between the neurons and the ensheathing glia, we photoconverted the neuron. We then quantified the ensheathment of the axonal projections by measuring the intensity profiles for both converted and unconverted *Tg(sox10:eos)* intensities that transect the axon and any associated glia (Fig. 3b, c). These intensity profiles revealed a decrease in the width of *Tg(sox10:eos)* unconverted^+^ ensheathing cells around the *Tg(sox10:eos)* converted axon in SU6656 treated animals, suggesting a lack of axonal ensheathment in these animals (Nichols and Smith, unpublished data). To determine the ensheathment of the neuronal cell bodies in both DMSO and SU6656 treated animals we rotated the images of the ensheathed neurons 90° to visualize the association of ensheathing cells with neurons in three dimensions. These rotated images revealed that the entire ganglion was ensheathed in both treatment groups (Fig. 3d, e), suggesting that ensheathment of the cell body is not affected by SU6656 treatment. These data are consistent with the hypothesis that the ensheathment of the neuronal soma is independent of axonal ensheathment mechanisms.

We sought to further characterize the ensheathment of the neuronal soma and the axon by determining the width of ensheathment in both neuronal compartments. It is possible that the ensheathment of the axon and neuronal soma are entirely independent and exhibit two distinct forms of ensheathment. However, it is also possible that similar biological principles underlie both processes so that similar ensheathment profiles are present in both neuronal compartments. To do this, we used *Tg(sox10:mcherry); Tg(ngn1:gfp)* animals to quantify intensity profiles transecting the axon and neuronal soma and calculated the ratios of the width of the *Tg(sox10:mcherry)* peak (ensheathing cells) to the width of the *Tg(ngn1:gfp)* peak. These ratios showed no significant difference between the ensheathment widths of the axon and neuronal soma (Fig. 3f, *p* = 0.3355, *n* = 10 DRG, 0.0853 ± 0.0839). This data suggest that similar ensheathment profiles are shared between the axon and the soma during early development. Later, axonal ensheathment likely expands as myelination occurs.

### Inhibiting axonal ensheathment leads to increased glia ensheathing the neuron cell body

We reasoned that the observed increase in ensheathing glia coverage in SU6656 animals was due to an axon-ensheathing cell returning to the ganglion after failed axonal ensheathment. Alternatively, failed axonal ensheathment could lead to increased proliferation of ensheathing cells. To test this, we first measured the area of the ensheathing glia at 72 hpf in both DMSO and SU6656 treated animals. These measurements showed an increase in the area of the ensheathing cells in SU6656 treated animals (Fig. 3g, *p* = 0.0006, *n* = 18 DMSO, *n* = 11 SU6656, DMSO: 156.923 ± 7.933 μm^2^, SU6656: 196.455 ± 13.301 μm^2^). To determine if this increase was due to an increase in size of individual glial cells or in the number of glial cells, we counted the number of glial cells that were associated with the neuronal cell body in both treatments. The SU6656 treated animals had, on average, one extra glial cell present in the ganglia compared to DMSO treated animals (Fig. 3h, *p* < 0.0001, n = 18 DMSO, n = 11 SU6656, DMSO: 2.278 ± 0.109, SU6656: 3.545 ± 0.207). We were also able to visualize this phenomenon using time-lapse imaging of axonal ensheathment. In SU6656 treated animals, a *Tg(sox10:eos)* unconverted^+^ cell (white arrow) travels dorsally along the nascent axon but returns to the soma before axonal ensheathment can occur (Fig. 3i). These two data sets are consistent with the hypothesis that if axonal ensheathment is inhibited during development, the glial cell that was to ensheath the axon returns back to the ganglia.

From these data, we hypothesized that the presence of an extra glial cell in the DRG could change the shape of entire ganglia. To do this, we determined the shape descriptors circularity, aspect ratio, roundness, and solidity for the ensheathing cells in both DMSO and SU6656 treated animals. We detected no significant change in the aspect ratio (Fig. 3j, *p* = 0.0970, n = 18 DMSO, n = 11 SU6656, DMSO: 2.249 ± 0.200, SU6656: 1.776 ± 0.125) or roundness (Fig. 3k, *p* = 0.0820, n = 18 DMSO, n = 11 SU6656, DMSO: 0.493 ± 0.0344, SU6656: 0.592 ± 0.0312) of the ensheathing cells, consistent with the conclusion that the extra cell did not lead to an elongation of the neuronal/ensheathing glia unit. However, both the circularity (Fig. 3l, *p* = 0.0057, n = 18 DMSO, n = 11 SU6656, DMSO: 0.622 ± 0.0244, SU6656: 0.506 ± 0.0290) and solidity (Fig. 3m, *p* = 0.0010, n = 18 DMSO, n = 11 SU6656, DMSO: 0.891 ± 0.0105, SU6656: 0.807 ± 0.0236) decreased in SU6656-treated animals. These shape changes are consistent with the conclusion that the extra glial cell from failed axonal ensheathment leads to a larger glial unit and an irregularly shaped, bulky ganglion. It is worth noting that our experiments do not distinguish whether SU6656 impacted these measurements cell-autonomously.

### Ensheathing progenitors exhibit space filling potential

**Fig. 4:**
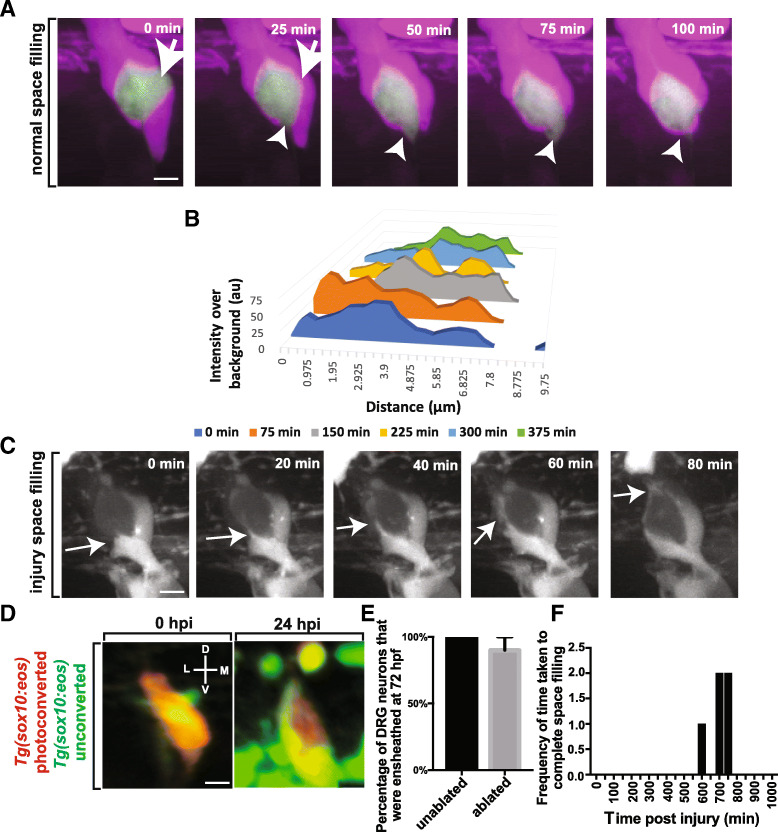
Cells ensheathing DRG neuron cell somas exhibit space filling throughout life. (**a**). Confocal z-projection images from a 24-h timelapse starting at 72 hpf of a *Tg(sox10:eos); Tg(ngn1:gfp)* with photoconverted ensheathing cells. White arrowhead and arrows denotes areas of neuronal soma that are re-ensheathed. (**b**). Intensity profiles transecting two approaching ensheathing processes every 30 min. (**c**). Confocal z-projection images from a 24-h timelapse starting at 48 hpf of a *Tg(sox10:eos)* zebrafish with a laser ablation of an ensheathing cell. White arrows denote the migrating ensheathing process. (**d**). Confocal 3D images of a *Tg(sox10:eos)* DRG with a photoconverted neuron and an ablated ensheathing cell at 0 and 24 hpi. D denotes dorsal, M denotes medial, V denotes ventral, and L denotes lateral. (**e**). Percent of DRG neurons that are ensheathed 24 hpi (*n* = 14 unablated DRG, n = 10 ablated DRG). (**f**). Histogram of the time after ablation that re-ensheathment of the neuron soma is completed (n = 5 DRG). Scale bar is 10 μm (**a**, **c**, **d**)

Our analysis thus far indicated that these ensheathing cells non-redundantly cover the neuronal cells in the DRG even despite considerable morphological changes. We hypothesized that these ensheathing cells could therefore exhibit continuous tiling potential which ensures cells' complete but non-redundant coverage of a target area. To first test this tiling hypothesis, we imaged the DRG during the proliferation of the ensheathing cells. In this experiment we observed that the individual neurons were continuously ensheathed. The only point where ensheathment was slightly disrupted was when an ensheathing cell divided and the neuron became momentarily and partially unensheathed (Fig. 4a). In this analysis over a 24-h period, we did not observe any neurons that became completely unensheathed. However, if these progenitors do exhibit continuous tiling behavior we would expect that even if a neuron becomes even momentarily unensheathed, the ensheathing cells would respond to eventually fully ensheath the neuron again. To test this, we imaged the projections of the ensheathing cells after partial unensheathment (Fig. 4a). In doing so, we were able to visualize ensheathing projections travel toward the unensheathed area of the soma. We further hypothesized that the stabilization of this re-ensheathment would recapitulate the first ensheathment paradigm where two ensheathing processes converged and retracted. To test this, we measured intensity profiles that transected the migrating processes as they approached each other every 75 min. These data showed cellular processes with a single intensity platform which 75 min later separated into two peaks (Fig. 4b, representative DRG chosen from 5 assayed DRG). These results are consistent with the hypothesis that these cells likely maintain continuous tiling behavior throughout life by recapitulating developmental ensheathment.

Given that multiple ensheathing cells and processes are present in the ganglia, it is possible that these cells exhibit this tiling behavior either equally or at different rates. To explore this possibility, we calculated the average velocity of each cell that responded to fill the empty space around the neuron. In responding to the partial unensheathment, responding processes within the same DRG exhibited differences in their speed at tiling the neuronal soma. These speeds ranged from 0.00515 μm/min to 0.0621 μm/min (mean ± SEM: 0.0286 ± 0.0059 μm/min). This data is consistent with the hypothesis that in normal tiling, the cells may not respond equally to partial unensheathment but instead one cell responds quickly and travels a further distance to fill space on the neuron.

To continue to provide mechanistic insight into the ability of these cells to tile the neuron we tested our hypothesis by manipulating the ensheathing cells experimentally to create an unensheathment event. Previous studies on tiling mechanisms have reported that the remaining ensheathing cells expand to cover the target area following cell ablation. To test the ability of DRG ensheathing cells to do this, we ablated one of the ensheathing cells and visualized the behavior of the remaining cells. To do this we created small ablations of individual ensheathing cells using our laser ablation system. In this paradigm, we created a 4 μm region of interest to ablate individual cells and then captured images every 5 min for 24 h (Fig. 4c, Additional file 2: Movie S2). First, we confirmed that the ablation resulted in an unensheathment event in the DRG by rotating the image of the ablated DRG 90° to view the ensheathment along the lateromedial axis of the animal at 0 and 24 h post injury (hpi). The resulting images revealed a neuron with little glial ensheatment following ablation (Fig. 4d), consistent with the idea that the ablation resulted in an unensheathment event. The same DRG 24 h later exhibited complete, three-dimensional ensheathment of the neuron (Fig. 4d). This change demonstrated that the remaining ensheathing cells responded to the ablation by filling the area on the neuron where the ablated cell existed. We scored that 90% of all DRG with an ablated glia had been re-ensheathed 24 hpi (Fig. 4e, *n* = 10 DRG).

Many possibilities, including cell divisions, glial volume expansion and rearrangement, could be responsible for the observed re-ensheathment. We hypothesized that re-ensheathment following injury recapitulated the developmental mechanism and resulted in the rearrangement of the remaining ensheathing glia. To test this hypothesis we visualized the movement of the ensheathing cells into the ablated site with time-lapse imaging. These movies showed that responding glia initiated projections that migrated to the ablated area where they converged, just as in developmental space filling. To further compare this experimentally induced space filling to that observed in development, we calculated the velocities of the responding glia. Just as in the developmental context, the injury-induced glial response led to asymmetric responses from the ensheathing cells where cells within the same DRG responded to the unensheathment with a wide variability of speeds ranging from 0.00493 μm/min to 0.0206 μm/min (mean ± SEM: 0.0149 ± 0.00155 μm/min). To gain a temporal understanding of the re-ensheathment events following injury, we recorded the times that the ensheathing cells required to re-ensheath the neuron following experimentally induced unensheathment. We found that the ensheathing cells required 679 ± 27.386 min after the injury event to re-ensheath the cell body (Fig. 4f, *n* = 5 DRG). Taken together, these data provide an understanding of ensheathment demonstrating re-ensheathment is performed by existing rearrangement of cells within the neuron/ensheathing cell unit. This is consistent with the hypothesis that ensheathing cells in the DRG exhibit continuous tiling capacity throughout life.

### Lack of ensheathment perturbs neuronal cell soma shape

**Fig. 5:**
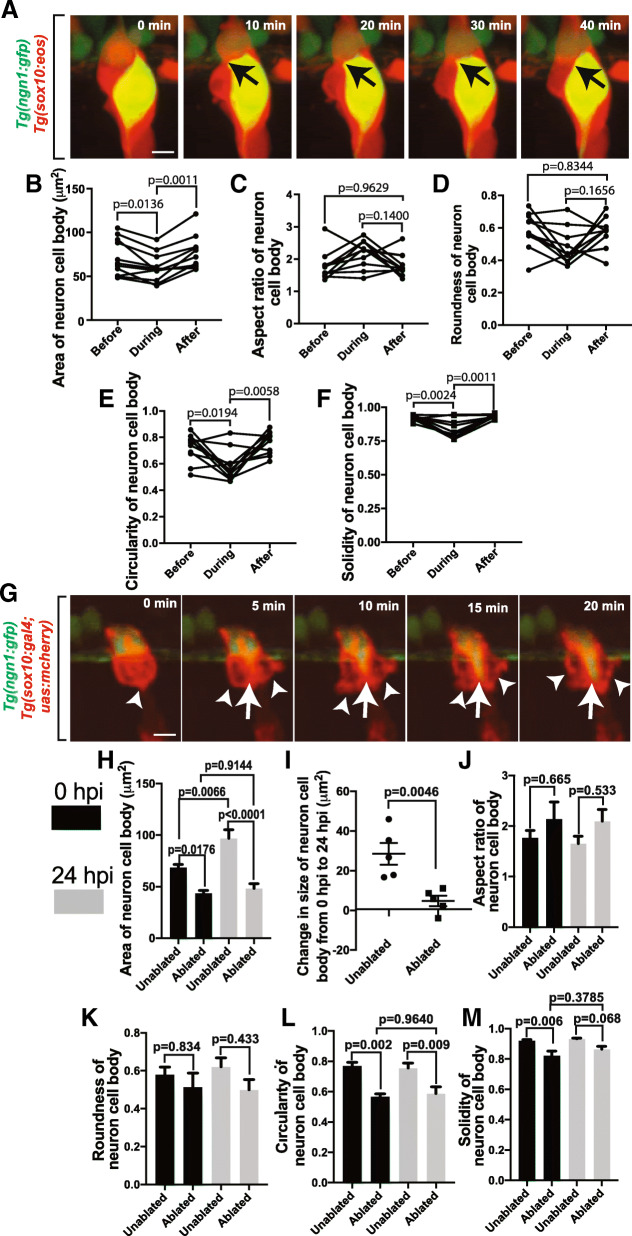
Perturbation of ensheathment impacts DRG neuron shape. (**a**). Confocal z-projection images from a 24-h timelapse starting at 72 hpf of a *Tg(ngn1:gfp), Tg(sox10:eos)* zebrafish with photoconverted *sox10*^*+*^ cells. Black arrows denote change in neuronal shape. (B-F). Graphs of area (**b**), aspect ratio (**c**), roundness (**d**), circularity (**e**), and solidity (**f**) of the neuron soma before, during, and after the division of an ensheathing cell (n = 11 neurons). (**g**). Confocal z-projection images from a 24-h timelapse starting at 48 hpf of a *Tg(ngn1:gfp), Tg(sox10:gal4; uas:mcherry)* zebrafish with photoconverted *sox10*^*+*^ cells and with an ablated ensheathing cell. White arrowheads denote responding ensheathing cells. White arrows denote the change in neuronal shape. (**h**). Area of the neuronal soma of DRG with ablated and unablated ensheathing cells at 0 hpi (black) and 24 hpi (gray) (n = 5 unablated DRG, n = 5 ablated DRG). (**i**). Change in the area of the neuronal soma in ablated and unablated DRG from 0 hpi and 24 hpi (n = 5 unablated DRG, n = 5 ablated DRG). (**j**-**m**). Graphs of the aspect ratio (**j**), roundness (**k**), circularity (**l**), and solidity (**m**) of the neuronal soma in ablated and unablated DRG at 0 hpi (black) and 24 hpi (gray) (n = 5 unablated DRG, n = 5 ablated DRG). (**b**-**f**) use a paired Tukey’s HSD test. (**h**,j-**m**) use an unpaired Tukey’s HSD test. (**i**) uses an unpaired Student’s *t*-test, Scale bar is 10 μm (**a**, **g**)

Neurons in the DRG exhibit a stereotypical round cell soma morphology and are positioned with precise topography in the ganglia [23, 33]. Due to their close association, these ensheathing cells have the potential to continuously impact neurons morphologically and physiologically. Previous research has demonstrated their physiological role [4, 5]. To investigate their potential role in neuronal morphology or spatial location in the ganglia we first investigated how neuronal shape changed in response to modulations in ensheathing cells. We hypothesized that if these ensheathing cells do continuously impact neuronal shape then even short perturbations to the ensheathment would impact neuronal shape. We first tested this possibility by visualizing neurons and ensheathing cells in *Tg(ngn1:gfp); Tg(sox10:eos)* animals from 48 to 72 hpf that have already extended processes. We specifically scored events in which the ensheathing cells proliferate, causing a partial unensheathment event, and then visualized the morphological behavior of the neurons (Fig. 5a). These images revealed a bulging of the neuronal soma during a partial unensheathment. To quantify this, we measured the area of the neuronal soma before, during, and after proliferation of the ensheathing cells. These measurements showed a dynamic decrease in area of the neuron during the perturbation of its glial sheath (Fig. 5b, *p* = 0.0136, *n* = 11 neurons, before: 71.51 ± 6.24 μm^2^, during: 59.94 ± 4.89 μm^2^) which then immediately increased back to its normal level following re-ensheathment (*p* = 0.0011, after: 76.43 ± 5.78 μm^2^). Using the shape descriptors for the neuron cell body, we were able to quantify the neuronal morphology before, during, and after proliferation of the ensheathing cells. The aspect ratio (Fig. 5c, *p* = 0.1400, n = 11 neurons, before: 1.78 ± 0.131, during: 2.174 ± 0.124, after: 1.82 ± 0.046) and the roundness (Fig. 5d, *p* = 0.1656, n = 11 neurons, before: 0.585 ± 0.0330, during: 0.478 ± 0.0323, after: 0.565 ± 0.0287) of neurons did not change during the proliferation of ensheathing cells. However, the circularity (Fig. 5e, *p* = 0.0194, n = 11 neurons, before: 0.722 ± 0.0315, during: 0.585 ± 0.0341) and solidity (Fig. 5f, *p* = 0.0024, n = 11 neurons, before: 0.924 ± 0.00575, during: 0.826 ± 0.0214) of the neuron decreased during glial proliferation. Both measurements (circularity: *p* = 0.0058, after: 0.766 ± 0.0256; solidity: p = 0.0011, after: 0.937 ± 0.00444) returned to their normal levels following the completion of glial proliferation, consistent with an overall decrease in area and the observed bulging of the neuron during a short perturbation of its glial sheath. These results are consistent with the conclusion that ensheathing cells may continuously provide a physical, restrictive force on DRG neurons.

To provide further mechanistic insight into this, we continued to test this hypothesis experimentally by ablating individual ensheathing cells and visualizing the morphological changes of the neuron. In these experiments, ablation of the ensheathing glia caused neurons to alter their morphology similar to the partial unensheathment from the proliferation of ensheathing cells (Fig. 5g). First, the ablation of an ensheathing cell led to a decrease in the area of the neuron shortly after the ablation compared to neighboring unablated control ganglia (Fig. 5h, *p* = 0.0176, *n* = 5 unablated DRG, n = 5 ablated DRG, ablated: 43.52 ± 2.97 μm^2^, unablated: 68.47 ± 3.01 μm^2^). This decrease in neuron size persisted following re-ensheathment and was still present 24 hpi (*p* < 0.0001 unablated DRG, n = 5 ablated DRG, ablated: 48.29 ± 4.56 μm^2^, unablated: 69.99 ± 8.34 μm^2^). To further explore this injury-induced perturbation of neuronal soma size, we quantified the change in the size of the soma from 0 to 24 hpi. Unablated control DRG neurons increased 26.53 ± 5.52 μm^2^ (Fig. 5i, n = 5 neurons) during that time period, while the neurons with an ablated glial cell increased only 4.77 ± 2.63 μm^2^ (n = 5 neurons, *p* = 0.0046). To continue to examine the neuronal morphological changes resulting from an unensheathment event, we quantified the shape descriptors for the neuronal cell bodies in DRG with ablated and with unablated ensheathing glia. Just as in the normal developmental partial unensheathment, the aspect ratio (Fig. 5j, *p* = 0.665, n = 5 neurons, unablated at 0 hpi: 1.767 ± 0.145, ablated at 0 hpi: 2.139 ± 0.336, unablated at 24 hpi: 1.654 ± 0.146, ablated at 24 hpi: 2.097 ± 0.230) and roundness (Fig. 5k, *p* = 0.834, n = 5 neurons, unablated at 0 hpi: 0.579 ± 0.0.0405, ablated at 0 hpi: 0.513 ± 0.0.0744, unablated at 24 hpi: 0.621 ± 0.0469, ablated at 24 hpi: 0.500 ± 0.0527) were not affected by unensheathment, suggesting that the neurons did not elongate. However, the circularity (Fig. 5l, p = 0.002, n = 5 neurons, unablated at 0 hpi: 0.769 ± 0.0245, ablated at 0 hpi: 0.567 ± 0.0188) and solidity (Fig. 5m, *p* = 0.006, n = 5 neurons unablated at 0 hpi: 0.921 ± 0.00662, ablated at 0 hpi: 0.822 ± 0.0298) both decreased in neurons with an ablated glia. The circularity of neurons with ablated ensheathing cells remained depressed 24 hpi (*p* = 0.009, unablated at 24 hpi: 0.756 ± 0.0330, mean ablated at 24 hpi ± SEM: 0.588 ± 0.0445). The solidity of these neurons slightly recovered to levels similar to that of DRG neurons without ablated cells (*p* = 0.068, unablated at 24 hpi: 0.933 ± 0.00530, ablated at 24 hpi: 0.864 ± 0.0185). The simplest explanation for this is that a larger unensheathment event following an injury to the ensheathing cells results in a persistent change to both the size and shape to the DRG neuron. Overall, these changes in neuron morphology in both the developmental and injury contexts are consistent with the hypothesis that ensheathing cells could provide continuous structural forces to maintain the morphology of individual DRG neurons.

**Fig. 6:**
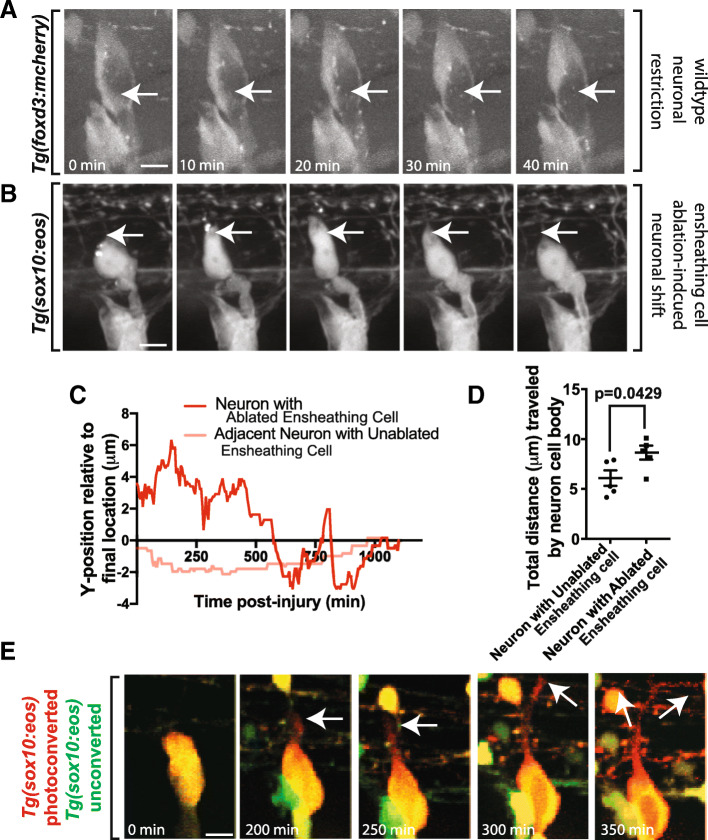
Extended perturbations of soma-ensheathing cells results in misplaced neurons. (**a**). Confocal z-projection images from a 24-h timelapse starting at 48 hpf of a *Tg(foxd3:mcherry)* zebrafish. White arrows denote the center of the neuronal soma. (**b**). Confocal z-projection images from a 24-h timelapse starting at 72 hpf of a *Tg(sox10:eos)* zebrafish with a laser ablation of multiple ensheathing cells. White arrows denote the original location of the dorsal apex of the DRG neuron. (**c**). Change in the y-position over time of a neuron in an ablated and unablated DRG. Y = 0 denotes the final location of the neuronal soma. (**d**). Total distance traveled by the DRG neuronal soma in an ablated and unablated DRG (n = 5 neurons). (**e**). Confocal z-projection images from a 24-h timelapse starting at 48 hpf of a *Tg(sox10:eos)* zebrafish with a photoconverted neuron and ablated ensheathing cell. White arrows denote the migration of the nascent axon. (**d**) use an unpaired Student’s *t*-test. Scale bar is 10 μm (**a**, **b**, **e**)

When DRG glial precursors are genetically ablated, the developing DRG becomes mislocalized at inconsistent locations along the dorsoventral axis of the animal [23]. Based on this observation and our data above that injury to ensheathing cells resulted in prolonged alterations in neuron size and shape, we hypothesized that prolonged unensheathment of neurons could have an impact on the location of the neuron within the ganglia. To address this possibility, we ablated multiple ensheathing cells and traced any movement of the neuron that they once surrounded. This quantification was done by measuring the center point of the neuron before and after the cells were ablated. In these experiments, we visualized that ablation of ensheathing glia first caused an immediate ectopic shifting of the neuron toward the area that was ablated (Fig. 6a, b). To determine if these neurons displayed a consistent direction of movement, we quantified the trajectory of the neurons following ablation and observed that neurons typically shifted either ventrally or dorsally (Fig. 6c). In these movements, the neurons did not move with a consistent velocity. Adjacent neurons without any ablated glia moved less. To further explore this injury-induced neuronal movement, we quantified the total displacement and velocities in these neurons. Neurons with ablated glia were displaced 8.476 ± 0.699 μm at a speed of 0.0077 μm/min (Fig. 6d, n = 5 neurons). These measurements were both greater than neurons in unablated DRG which traveled an average distance of 6.0 ± 0.776 μm at a speed of 0.0054 μm/min (Fig. 6d, *p* = 0.0429, *n* = 5 neurons). These tracings are consistent with the possibility that prolonged unensheathment of DRG neurons could cause not only momentary morphological changes (Fig. 5) but also shifting of the neurons within the ganglia.

Given that ablation of ensheathing cells resulted in misplaced DRG neurons, we next tested if ablation of ensheathing cells resulted in axonal pathfinding defects. However, we did not observe any obvious pathfinding defects. The axon initiated and traveled dorsally, entering the spinal cord at a typical DREZ location (Fig. 6e).

### Proliferation of neurons corresponds with expansion of progenitors

**Fig. 7:**
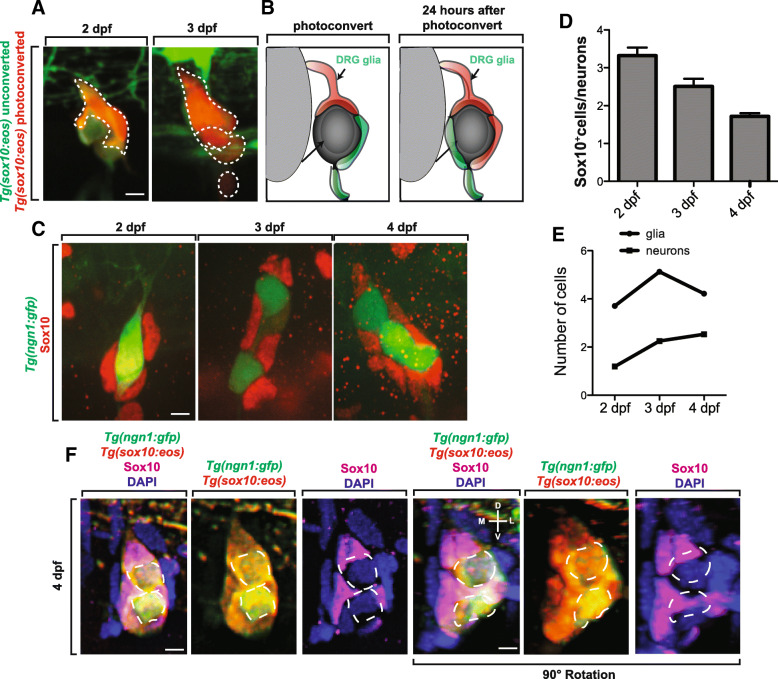
Proliferation of ensheathing cells is correlated with DRG sensory neuron expansion. (**a**). Confocal z-projection images of a *Tg(sox10:eos)* zebrafish with a single ensheathing cell photoconverted at 2 dpf. Images were taken at 2 and 3 dpf. Dashed outlines denote *Tg(sox10:eos)* photoconverted^+^ cells. (**b**). Schematic summary of the use of photoconversion in the proliferation of ensheathing cells. (**c**). Confocal z-projection images of *Tg(ngn1:gfp)* zebrafish stained with Sox10 at 2–4 dpf. (**d**). Ratio of the number Sox10^+^ cells to the number of neurons in a DRG at 2–4 dpf (*n* = 16 DRG). (**e**). Number of Sox10^+^ cells and neurons present in the DRG at 2–4 dpf (*n* = 16 DRG). (**f**). Confocal z-projection images of a *Tg(ngn1:gfp); Tg(sox10:eos)* 4 dpf zebrafish with photoconverted Eos and stained with Sox10 and DAPI. Bottom row is rotated 90°. Dashed outlines denote neuronal somas. D denotes dorsal, L denotes lateral, V denotes ventral, and M denotes medial. Scale bar is 10 μm (**a**, **c**, **f**)

Since these ensheathing cells are present during early ganglia development, we sought to identify if they represented a terminally defined cell-type or if they were a progenitor population. To first test the progenitor possibility of these ensheathing cells, we imaged *Tg(sox10:eos)* animals at 2 dpf and photoconverted a single ensheathing cell. Twenty-four hours later, we observed multiple *Tg(sox10:eos)* photoconverted cells in the DRG, suggesting that the ensheathing cells actively divide like a progenitor population (Fig. 7a, b) as previously described [37]. To further explore this possibility, we quantified whether they expanded as the neuron number increased. To do this, we imaged the DRG in *Tg(ngn1:gfp); Tg(sox10:eos)* animals at two, three, and four dpf and photoconverted the Eos in the entire animal (Fig. 7c). In these images, we quantified the ratio of *sox10*^*+*^ cells and neurons at each time point. The ratios steadily decreased at each time point (Fig. 7d). To further expand this analysis, we quantified the number of ensheathing cells and neurons present in the DRG at each time point (Fig. 7e). From 2 to 4 dpf, the number of neurons increased by one on average. The number of glia increased by one from 2 to 3 dpf but then decreased by one by 4 dpf. Overall, these data are consistent with the idea that the proliferation of the ensheathing cells corresponds with an increase in sensory neurons and that the new neurons could arise from *sox10*^+^ ensheathing progenitor cells.

We next sought to gain a spatial understanding of DRG expansion. We hypothesized that ensheathing cells could ensheath multiple neurons during DRG expansion. However, it is also possible that neurons could be individually encased by individual cells. To test these possibilities, we used *Tg(ngn1:gfp); Tg(sox10:eos)* fish and photoconverted Eos at 4 dpf. After photoconverstion, animals were fixed and stained for Sox10 and DAPI. Images of these animals revealed that each neuron was individually encased by multiple ensheathing cells (Fig. 7f). While it is difficult to determine exactly how many cells participate in the ensheathment of an individual neuron, more than one cell nuclei that associated with processes that ensheath individual neurons at 4 dpf can be visualized, a result that is consistent with recent investigations into adult DRG ensheathment by mature satellite glia [9].

### Ensheathing cells respond to neuronal injury

**Fig. 8:**
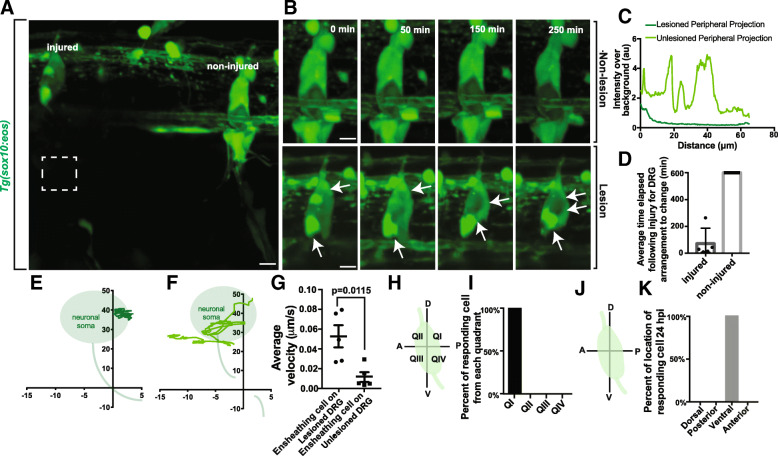
Ensheathing cells respond to peripheral neuronal injury. (**a**). Confocal z-projection image of a *Tg(sox10:eos)* zebrafish with a DRG with a peripheral lesion and an adjacent non-lesion DRG. White box denotes site of lesion. (**b**). Confocal z-projection image of a 24-h timelapse starting at 72 hpf in a *Tg(sox10:eos)* zebrafish. Top images denote a non-lesion DRG, and bottom images denote a DRG with a peripheral lesion. White arrows denote the movement of ensheathing cells. (**c**). Intensity profile of lesioned and non-lesioned peripheral axons. (**d**). Time elapsed after lesion for rearrangement of ensheathing cells in lesioned and non-lesioned DRG (n = 5 lesion DRG, n = 5 non-lesion DRG). (**e**-**f**). Plots of ensheathing cells following axonal lesion on non-lesioned (**e**) and lesioned (**f**) neurons. Shaded gray areas denote location of the neuron. (0,0) denotes the center of the peripheral axon. (**g**). Average velocity of ensheathing cells following injury on lesioned and non-lesioned neurons (n = 5 lesion DRG, n = 5 non-lesion DRG). (**h**). Schematic compass of DRG neuron into quadrants. D denotes dorsal, P denotes posterior, V denotes ventral, A denotes anterior. (**i**). Percentage of responding cells that originate in each quadrant of the neuron (n = 5 DRG). (**j**). Schematic compass of DRG neuron by anatomical descriptor. D denotes dorsal, P denotes posterior, V denotes ventral, A denotes anterior. (**k**). Percentage of responding cells that stabilize in each portion of the neuron (n = 5 DRG). (**g**) uses an unpaired Student’s *t*-test. Scale bar is 10 μm (**a**, **b**)

Since these ensheathing cells are closely associated with sensory neurons from as early as we can visualize, we hypothesized that they would quickly respond to changes in neuronal homeostasis [10]. Although this phenomenon has been demonstrated, the temporal dynamics of this have not been thoroughly examined. To test this, we imaged DRG neurons that were axotomized distally in the periphery and visualized the response of the ganglia cells to that injury. Using *Tg(sox10:eos)* animals which label cells that ensheath the DRG neurons we performed an 8 μm axotomy injury approximately 100 μm from the ganglia and then collected images every 5 min for 10 h. In each image, we had an experimental-injured and a control non-injured sensory nerve (Fig. 8a). We first confirmed that our laser parameters created a complete transection of the peripheral nerve by imaging *Tg(sox10:eos)* intensity profiles of the length of the lesioned axon 1 h following injury and visualized a lack of fluorescent signal along the nerve (Fig. 8c).

In these animals we visualized that 100% of ganglia with injured peripheral nerves responded by re-arranging ensheathing cells around the neuronal soma (Fig. 8b, Additional file 3: Movie S3, Additional file 4: Movie S4). In control, non-injured, ganglia that were adjacent to the injured nerves we did not visualize this dynamic; within the 10-h period of imaging, 0% of ganglia associated with non-injured nerves re-arranged. We took this analysis further to dissect the dynamics of re-arrangement in order to investigate the speed of ganglia response to peripheral injury. Injured nerves induced a ganglia cell re-arrangement in 72.6 ± 48.22 min with the majority responding within the first hour (Fig. 8d, *n* = 5). These responses occurred in the ganglia 100 μm from the injury site. These results are consistent with the hypothesis that the ensheathing cells of the ganglia can respond rapidly to peripheral injury of those neurons.

To expand our understanding of the injury response, we traced the movements of the responding glia in DRG with lesioned axons as well as adjacent unlesioned DRG. Ensheathing cells in lesioned DRG dynamically traversed the entire ganglia, while ensheathing cells in unlesioned DRG remained in their original location (Fig. 8e, f). Further, we calculated the velocities of the ensheathing cell projections in both lesioned and unlesioned DRG. These cells responded to the lesion with a velocity of 0.0519 ± 0.0113 μm/min (Fig. 8g, n = 5 DRG), while cells on unlesioned ganglia were largely stationary (n = 5 DRG, *p* = 0.0115, 0.0117 ± 0.00492). Since the injury was consistently created in the same portion of the neuron, we hypothesized that the glial response may be similar following each injury. To do this, we determined the location of the cells that respond to the peripheral injury by quantifying which quadrant of the ganglia the responding cell originated. We found that 100% of the responding cells on lesioned DRG originated in the dorsal, posterior quadrant of the ganglia (Fig. 8h, i, n = 5 DRG). Given this highly consistent spatial response to the injury, we also quantified the final location of the responding cell. We hypothesized that the responding cell may travel to the sight of the injury. Instead, 100% of the responding cells eventually ended their migration at the ventral apex of the neuron cell body where the injured neurite originated (Fig. 8j, k, n = 5 DRG). These data suggest that ensheathing cells have a strikingly consistent and choreographed response to peripheral axonal injury; they migrate to the extension site of initiation of the injured axon.

Given the effects of unensheathment can have on the neuron and the apparent rearrangement of cells in the ganglia following injury, we next asked if neuronal soma shape was momentarily altered during the injury response. To ask this, we measured the area and shape descriptors for the neuronal cell bodies shortly after the injury and 24 h later. We did not detect any changes in area (*p* = 0.6830, n = 5 unlesioned neurons, n = 5 lesioned neurons, unlesioned at 0 hpi: 61.199 ± 9.688 μm^2^, lesioned at 0 hpi: 73.937 ± 15.863 μm^2^, unlesioned at 24 hpi: 76.472 ± 12.843 μm^2^, lesioned at 24 hpi: 83.465 ± 13.101 μm^2^), circularity (*p* = 0.8453, n = 5 unlesioned neurons, n = 5 lesioned neurons, nonlesioned at 0 hpi: 0.775 ± 0.0600, lesioned at 0 hpi: 0.768 ± 0.0325, nonlesioned at 24 hpi: 0.765 ± 0.0595, lesioned at 24 hpi: 0.796 ± 0.0380), aspect ratio (*p* = 0.2491, n = 5 unlesioned neurons, n = 5 lesioned neurons, nonlesioned at 0 hpi: 1.438 ± 0.188, lesioned at 0 hpi: 1.868 ± 0.125, nonlesioned at 24 hpi: 1.536 ± 0.131, lesioned at 24 hpi: 1.713 ± 0.168), roundness (*p* = 0.2040, n = 5 unlesioned neurons, n = 5 lesioned neurons, nonlesioned at 0 hpi: 0.739 ± 0.0857, lesioned at 0 hpi: 0.545 ± 0.0357, nonlesioned at 24 hpi: 0.671 ± 0.0571, lesioned at 24 hpi: 0.608 ± 0.0650), or solidity (*p* = 0.6562, n = 5 unlesioned neurons, n = 5 lesioned neurons, nonlesioned at 0 hpi: 0.914 ± 0.143, lesioned at 0 hpi: 0.930 ± 0.0106, nonlesioned at 24 hpi: 0.918 ± 0.185, lesioned at 24 hpi: 0.921 ± 0.0172). These measurements suggest that the ensheathing glia exhibit a highly-coordinated response throughout the ganglia following peripheral injury that mirrors developmental expansion to ensure continued ensheathment of the neuronal cell body as one cell migrates across the ganglion. Only during longer pathological unensheathment events does neuronal morphology become altered. Together, these data support the hypothesis that during the lifespan of the DRG, the neuron and its dynamic ensheathing cells are intimately connected and plastic.

## Discussion

Ensheathment of neuronal cells is critical to proper formation and function of complete nerves. Using single-cell photoconversion and time lapse imaging in intact living vertebrates, we demonstrate that the ensheathment of DRG neuronal cell somas occurs during neuronal differentiation, a distinct event from axonal ensheathment. We show that these ensheathing cells must rearrange to allow for the initiation of neurites. During development they also exhibit tiling behavior. Perturbations of the soma ensheathment causes changes in neuronal soma morphology and positioning. Using laser induced lesions of the distal peripheral axon, we show that these ensheathing cells respond quickly to neuronal injury in a choreographed manner. Together, our data suggest that ensheathing cells of the DRG neuronal cell soma are closely associated with the neuron starting shortly after initial neuronal differentiation and persist throughout the life of the neuron to maintain stereotypical ganglia positions.

### DRG cells exhibit a neural niche

The proliferation of neural progenitor populations is necessary for the establishment and creation of a complete neuronal circuit [46]. These progenitor populations are often confined to small areas, called neural niches, where the production of neural precursors can be tightly controlled and regulated [47]. Previous studies have shown that cortex glia, a *Drosophila* cell type that ensheathes neuronal cell somas in adult brains, ensheath distinct neural progenitor populations in neural niches [32]. This ensheathment persists throughout the proliferation of the neural progenitors. Our data suggest that the development of the dorsal root ganglia can serve as an analogous model for such neural niches. We demonstrate that ensheathment of the DRG neuronal cell soma occurs shortly after neuronal differentiation and persists throughout the expansion of the DRG. Further, these data demonstrate that the ensheathing cells of the DRG serve as a progenitor population for DRG neurons [37], consistent with neuronal progenitor differentiation in classical neural niches. In fact, the fate of progenitor populations in neural niches and the DRG may even depend on the level of ensheathment of a daughter cell by dictating expression of neuronal markers. As a result, future studies on the precise development of the DRG in the context of ensheathing cells may yield valuable insights into the dynamics of neural niches and neuronal differentiation.

In addition, glial ensheathment of distinct portions of a mature neuron is critical to the establishment of a functional nervous system [10, 48]. While many previous studies have examined the mechanism of axonal ensheathment, few have explored the process of neuronal cell soma ensheathment. Our data helps fill this gap by visualizing the ensheathment of DRG neuronal somas in vertebrates. We elucidate the close association between the ensheathing cells and the neuron throughout its maturation. Studies in *Drosophila* have demonstrated the potential for cells ensheathing the cell soma to interact with and support the neuron which they ensheath. For example, expression of a (DE)-cadherin dominant negative in *Drosophila* cortex glia, which ensheath neuronal cell bodies, have been shown to lead to misplaced neural progenitors and neuronal somas as well as disrupt neuron morphology and neurite trajectories [18]. Here, we provide a step wise visualization of the ensheathment of such vertebrate neuronal somas.

### Soma ensheathing cells interact with axons

In a mature DRG, differentiated satellite glial cells ensheath the sensory neurons located in the ganglia [10]. Interestingly, co-cultures of mature satellite glial cells and neurons lead to inhibited dendrite formation [49]. Our data suggests that the precursors to satellite glial cells are intimately involved in neuronal maturation and morphology, including neurite extension. Given this and the formation of “glial horns” during neurite initiation, the ensheathment of the neuron cell body is likely critical to maturation of the neuron and its neurites. Coupled with increasing evidence for the role of glia in the initiation of neurites [15], our data suggest that rearrangement of the ensheathing DRG cells may provide a substrate to allow for the initiation or extension of axonal projections. Further, we demonstrate that the initial ensheathing cell of the sensory pioneer axon are produced in the ganglia by cells that ensheath the cell soma. This finding complements recent investigations seeking to characterize satellite glia which identified myelinogenic capabilities of mouse satellite glia in culture as well as robust transciptomic similarities between satellite glia and mature Schwann cells [9]. Our imaging of DRG development in zebrafish are consistent with these hypotheses. The intimate associations of the ensheathing cells with the DRG neurons from as early as we can delineate suggests they could have a profound influence on neuronal development and homeostasis. But to date, neuronal development and homeostasis are often investigated in the absence of ensheathing cells. Future studies testing these hypotheses may yield important insights in axonal morphology, nerve assembly, and axonal compartmentalization.

### Forces that dictate neuronal cell shape and location

Further, our data suggest that neuronal soma ensheathment likely plays a conserved function in dictating the size and shape of the soma in development. In zebrafish mutants of Sox10, a gene required for glial differentiation, ensheathing cells of the DRG are absent and the DRG are mislocalized [23]. These data are consistent with the hypothesis that ensheathing cells also contribute to the positioning of the DRG. We also observe that the neuronal soma immediately bulges and shifts toward a site of disrupted ensheathment. The totality of these data, in conjunction with previous studies, point to a conserved role of continuous mechanical forces on the neuronal soma from the ensheathing cell membranes imposing a circular shape and potentially precise position on the neuron within the ganglia [17, 18, 23, 32]. The release of this restraining force in unensheathment events likely causes the neuron to accelerate toward ablated area much like a compressed spring.

The DRG has a precise and stereotypical spatial and temporal arrangement of neurons where specific neuronal subtypes localize to distinct regions within the ganglia [33]. In addition to neurons, ensheathing cells in the DRG must also continuously produce other cell types such as Schwann cells and melanocytes which then traverse around the ganglia to leave for their respective target areas [34, 50, 51]. Throughout each of these proliferation events, DRG neurons retain their precise location in both development and adulthood. As a result, a continuous mechanism must be employed in the ganglia to anchor DRG neurons in their locations throughout cell proliferation events while allowing differentiating cells to migrate away from the DRG. The data presented here suggest that the tiling behavior of soma-ensheathing cells could continuously help maintain precise shape and arrangement of DRG neurons by providing forces on the neuronal soma. Following a prolonged, injury-induced unensheathment event, the DRG neuron immediately bulges and becomes displaced from its original position, like an expanding spring. However, during homeostatic proliferation of ensheathing cells and partial unensheathment events, the neuronal movement is less pronounced. A greater mislocalization of DRG neurons was caused by complete, genetic ablations of ensheathing progenitors [23]. Together, this is consistent with continuous role of ensheathing cells to physically provide forces on DRG neurons throughout paradigmatic development. In addition, physical contacts between DRG cell types have been suggested as regulators of cell fate decisions between glia and neurons as well as between different types of neuronal subtypes [23, 33, 50]. Taken together with our data, this suggests that the precise forces on neurons by ensheathing cells may aid in the development of the diversity of somatosensory cell types.

Unfortunately, the physiological effects of changes to the shape of neuronal somas are currently unknown. Data comparing the morphology and electrophysiology of CA1 and CA2 hippocampal neurons in *Proechimys* rodents suggests that smaller cell somas are correlated with decreased electrical resistance and a longer latency period [52]. However, these recordings were taken from healthy and fully differentiated neuron populations. Cell soma size also correlates with axonal caliber [53]. Studies show axonal caliber impacts ensheathment and myelination, pointing to a hypothesis that neuronal size is important in the nervous system [54, 55]. Regardless, given that our data indicate that re-ensheathment itself is not sufficient to restore neuronal morphology following prolonged unensheathment, the importance of soma shape in neuron electrophysiology, although beyond the scope of this paper, should be investigated, especially given the possibility of unensheathment in disease states.

### Ensheathing cells respond to injury

Given the close proximity of ensheathing cells to axonal injury, they represent an important cell type that could respond to neural injury. Previous studies have shown that many ensheathing glial subtypes exhibit tiling behavior to maintain neuronal ensheathment following injury [16]. Our analysis of the space filling behavior by ensheathing processes in both normal development and injury states consistently demonstrated an asymmetric response to unensheathment by individual cells (Fig. 4). This response suggests that specific subpopulations of ensheathing glia, at least during development, may be hypervigilant to unensheathment events. We also demonstrate that ensheathing cells respond to modulated ensheathment through tiling behavior throughout the life of the DRG. Further, we show that neuronal soma-ensheathing cells display a consistent and choreographed response to neuronal injury to the distal axonal segment by migrating to the initiation site of the injured neurite. This response does not disrupt the ensheathment or morphology of the neuron, suggesting a coordinated space-filling response by all ensheathing cells following neuronal injury. As a result, our data point to DRG ensheathing glia as a population that can detect and respond to neural injury, a behavior also seen in mature satellite glial cells [10]. This observation is also consistent with previous reports in *Drosophila* where cortex glia respond to injury and phagocytize neural debris [16]. Previous studies have also demonstrated that some DRG cell types are pluripotent stem cells that can differentiate into terminal glia, or even skin melanocytes, after migrating down the peripheral nerve to the skin [34]. Given the specific choreographed response by ensheathing glia from a consistent and specific neuronal domain following neural injury, it is possible that the responding cell we identified retains a stem-like quality in order to coordinate a cellular response to neuronal injury. Coupled with the hypervigilance of some cells to unensheathment, the cells that ensheath DRG neurons could form a heterogeneous population with specific cells primed to respond to various, yet specific disruptions to neuronal homeostasis. Future research will dissect this important topic.

## Conclusions

While many studies have been devoted to the mechanisms of axonal ensheathment and its role in neuronal homeostasis, mechanisms of ensheathment of neuronal somas has remained elusive. Here we used single-cell photoconversion to visualize the process of soma ensheathment in zebrafish dorsal root ganglia. Taken together, our data point to the importance of neuronal soma ensheathment in nerve development as early as neuronal differentiation. The close association of the ensheathing cells with the maturing neuron point to cross-talk as a possible important facet of nerve assembly. Elucidating these associated cues, as demonstrated by recent studies, could yield valuable insight into neural niches, neural injury responses and the diversity of soma-ensheathing cells.

## Additional files

Additional file 1: **Movie S1.** Ensheathment of neuronal progenitors occurs soon after neuronal differentiation. Excerpt from a 24-h time lapse of a *Tg(sox10:eos)* animal with a photoconverted neuronal progenitor from 48 to 72 hpf. (Left) A *Tg(sox10:eos)* unconverted cell wraps projections around the neuronal progenitor before neurite extension. (Right) Merged image of *Tg(sox10:eos)* converted (neuronal progenitor) and unconverted (ensheathing cell). Green arrows denote ensheathing projections. Dark green arrow denotes merged ensheathing projections. Frames in the video were captured every 5 min, and the video plays at 10 frames per second. Supplements Fig. 1d. (MOV 4824 kb)

Additional file 2: **Movie S2.** Soma-ensheathing cells exhibit tiling behavior during injury. Excerpt from a 24-h time lapse of a *Tg(sox10:eos)* animal with a photoconverted neuronal progenitor from 48 to 72 hpf following a laser ablation of an ensheathing cell. (Left) *Tg(sox10:eos)* unconverted cells respond to the unensheathment of the neuronal soma by sending projections to restore soma ensheathment. (Right) Merged image of *Tg(sox10:eos)* converted (neuron) and unconverted (ensheathing cell). Green arrows denote ensheathing projections. Dark green arrow denotes merged ensheathing projections. Frames in the video were captured every 5 min, and the video plays at 10 frames per second. Supplements Fig. 4e. (MOV 6449 kb)

Additional file 3: **Movie S3.** Ensheathing cells are stationary in the absence of neuronal injury. Excerpt from a 24-h time lapse of a *Tg(sox10:eos)* animal from 72 to 96 hpf. The imaged DRG is adjacent to a DRG neuron with peripheral axonal injury at 72 hpf. Red arrow denotes an ensheathing cell. Frames in the video were captured every 5 min, and the video plays at 10 frames per second. Supplements Fig. 8b. (MOV 931 kb)

Additional file 4: **Movie S4.** Ensheathing cells dynamically remodel following distal axonal injury. Excerpt from a 24-h time lapse of a *Tg(sox10:eos)* animal from 72 to 96 hpf. The imaged DRG neuron suffered distal axonal injury at 72 hpf. Red arrow denotes an ensheathing cell. Frames in the video were captured every 5 min, and the video plays at 10 frames per second. Supplements Fig. 8b. (MOV 1382 kb)

## Abbreviations

CNS: Central nervous system
DMSO: Dimethyl sulfoxide
dpf: Days post fertilization
DREZ: Dorsal root entry zone
DRG: Dorsal root ganglia
hpf: Hours post fertilization
hpi: Hours post injury
